# Accuracy evaluation of smartphone-based GNSS position and speed tracking for ski-slope and safety management

**DOI:** 10.1371/journal.pone.0327896

**Published:** 2025-08-13

**Authors:** Davide Petrella, Lynn Ellenberger, Matthias Gilgien

**Affiliations:** 1 Department of Movement Sciences, KU Leuven, Leuven, Belgium; 2 Swiss Council for Accident Prevention BFU, Bern, Switzerland; 3 Department of Physical Performance, Norwegian School of Sport Sciences, Oslo, Norway; 4 Centre of Alpine Sports Biomechanics, Alpine Health and Innovation Foundation, Udligenswil, Switzerland; ASPIRE Academy for Sports Excellence, QATAR

## Abstract

Smartphones with integrated global navigation satellite system (GNSS) functionality are increasingly used in various apps beyond communication, including positioning, navigation, and tracking. This study explores the potential of smartphone GNSS data to improve ski slope safety through motion data analysis. Apps such as iSKI, Skitude, Slopes, and Strava measure speeds, distances, and altitude differences, generating valuable data on skiers’ movements. These data help ski resorts in planning and accident prevention by identifying high-risk areas based on movement patterns. We compared the accuracy of position and speed data from four apps across four smartphone models (two Android and two iOS) against a differential GNSS (dGNSS) reference system. Data were collected at two ski resorts during the winter of 2022/23, with smartphones recording at 1 Hz and dGNSS at 50 Hz. Analysis focused on downhill runs, excluding initial recording phases and vertical position data. Accuracy was assessed by calculating the Euclidean distance between the time-synchronized smartphone data and dGNSS reference data. High-end smartphones provided more accurate position data, with an average error of approximately 4 m, compared to 6 m for low-end models. Speed data were reliable across all devices, with an average error <1.9 km/h. However, accuracy diminished with increasing speeds and varied based on location-specific environmental factors. Thus, although smartphone position data can evaluate non-exact position-dependent parameters, such as slope utilization and user density, more precise systems, such as dGNSS, are necessary for exact position-dependent evaluations. Speed data derived from cleaned position data are reliable for estimating skier speeds, and data from different apps can be combined if consistent calculation methods are used. Future advances in smartphone technology are expected to enhance data accuracy. Recommendations include using smartphone data in open terrain for better accuracy and exercising caution when interpreting absolute position data for accident prevention or other context-specific analyses.

## Introduction

Smartphones serve numerous purposes in today's world beyond communication. One increasingly popular feature is positioning via the built-in global navigation satellite system (GNSS), which is typically used for localization, navigation, and tracking. GNSS-based apps are particularly popular in sports, such as cycling and running. The apps allow users to track their activities, measure their performance and training load, and motivate themselves through features and by connecting with other users. Among winter sports, recreational alpine skiing and snowboarding stand out as key areas of use for this technology. Apps used in these sports provide detailed map guidance, run tracking, and distance and speed measurement, as well as entertainment features, such as challenges. Most apps rely on GNSS and air pressure sensors to monitor skier and snowboarder activities.

With large user bases, app providers can aggregate GNSS tracking data to analyze population-level behaviors, including location, timing, and dynamics (e.g., speed, breaks). These data help describe skier and snowboarder movements within ski resorts in relation to the infrastructure, time, and weather, offering valuable insights for planning, management, and optimization. For example, information on the spatio-temporal movement patterns of skiers can help in planning lift capacity, optimizing slope utilization, and managing visitor flows more efficiently. Some resorts already offer dedicated apps to collect and utilize such data. With progressive digitalization, more ski resorts are likely to adopt similar technologies.

Motion data from snow sport participants are also of great interest from the perspective of accident prevention. The movement patterns of skiers in relation to, for example, slope width, usage frequency, and terrain type likely influence slope safety but remain under-researched. In road traffic, similar data collected by the fitness app Strava are already being used to plan cycle routes [1]. This approach could be adapted to winter sports to enhance safety.

To exploit the potential of aggregated ski and snowboard data for optimizing ski resorts and on-slope safety, it is essential to understand the possibilities and limitations of such data. The limitations of using smartphone GNSS data to describe skiing as a dynamic, continuous process in space and time, with the skier represented as a point mass, are influenced by the accuracy and continuity of the position and speed data. Although the point mass motion of alpine skiing has been determined successfully and validated with dedicated industrial and consumer grade GNSS units [2–6], the validity of smartphone GNSS, which may have reduced accuracy due to form factor and battery power constraints compared to the dedicated GNSS units, remains unknown. However, ongoing advances in software, satellite systems, and correction services may improve position accuracy [7,8].

This raises two important questions about accuracy – how the dynamic application and the sensor type of the smartphone affect the accuracy.

First, regarding snow sport-specific accuracy, the position tracking performance of the smartphone has been validated for static use and various human locomotion modes [9–13], but its accuracy for dynamic applications, such as alpine skiing and snowboarding, remains uncertain. Previous studies have shown that the shading of GNSS signals by the mountains surrounding ski resorts has a significant effect on the accuracy of even industrial-grade GNSS when used in dynamic alpine skiing applications [14]. In addition, the accuracy of dynamic GNSS applications is lower than the accuracy of corresponding static applications [15]. Furthermore, frequent changes in direction caused by turning in alpine skiing impact accuracy [16]. Therefore, the negative effects of limited GNSS signal availability and the dynamics of alpine skiing and snowboarding are expected to compromise the accuracy of smartphone-based GNSS, and application-specific validation is necessary, also for smartphones.

Second, regarding device and app variability, whether smartphone models (high- and low-end Android and iOS) and smartphone apps designed to track alpine skiing differ in their GNSS signal-based position and speed tracking accuracy is unknown. These are important questions, especially if data from a skiing population is gathered for analysis by sourcing from different types of smartphones and apps, as differences in their accuracy could have an impact on the validity of the analysis.

In this study, we investigated the accuracy of smartphone GNSS-based position and speed measurements in recreational alpine skiing at two representative ski resorts for four different smartphones and four different apps.

## Materials and methods

### Measurement protocol

Data were collected during two days in Davos, Jakobshorn (WGS84 coordinates: 46.773, 9.849; Davos Day1 & Davos Day2) and one day in Zermatt, Trockener Steg (WGS84 coordinates: 45.972, 7.722), Switzerland in 2023. Two recreational skiers (Skier A & Skier B) were equipped with an industrial grade differential GNSS (dGNSS) and several smartphones. The dGNSS system served as the ground truth for position and speed tracking, whereas the smartphones were carried for evaluation of their accuracy in measuring position and speed. The smartphones included two iOS devices and two Android devices, representing both high-end and low-end models commonly used by the general population. Low-end devices were also tested in battery saver mode for a portion of the time to assess its effect on accuracy. Tracking was conducted simultaneously using four skiing apps (Strava, iSki, Slopes, Skitude) to assess their effect on tracking accuracy. Pretesting confirmed that the simultaneous use of these apps does not cause interference. The dGNSS device was carried on the skier's back with the GNSS antenna at approximately the fourth thoracic vertebra (T4), representing the position of the skier as a point mass. Smartphones were carried in typical locations on the body, namely in the pockets of the ski jacket and pants, with only one device per pocket, causing a physical offset of several decimeters between the dGNSS position and the smartphones. The GNSS tracking was started at least 15 minutes prior to the start of the skiing measurement to allow for the download of broadcast ephemerides. The ground truth dGNSS system comprised a GNSS receiver (AsteRx-i S, Septentrio, Leuven, Belgium) and GNSS antenna (TW7972, Tallysman, Ontario, Canada), which collected multi-frequency and multi-constellation GNSS raw data at 50 Hz. A GNSS base station (Altus NR3, Septentrio, Leuven, Belgium) was positioned <1 km from the slope and logged GNSS raw data at 50 Hz for dGNSS post-processing, providing high-resolution positional accuracy. The sampling frequency of the smartphones varied depending on the specific application being used. Accuracy was expected to be on the scale of meters, so the ground truth was substantially more accurate and valid for the assessment. Only the fractions of the collected data corresponding to actual downhill skiing were included in the analysis. Data segments related to lift transportation, static situations, and other non-skiing activities were excluded using an algorithm. Table 1 summarizes the data included in the analysis, detailing the distance, altitude drop, speed, and duration of the activity. These metrics were derived from the dGNSS ground truth data. This study was approved by the ethics committee at the Norwegian School of Sport Sciences (number 252–101122), and the Norwegian Centre for Research Data, and was conducted according to the Declaration of Helsinki. The participants were recruited in the time period 05/01/2023 and 12/01/2023 and gave informed written consent to participate in the study that was documented on 12/01/2023 by e-mail.

**Table 1:** Summary of skiing activity included in the study, showing distance, altitude drop, average and maximum speed, and duration of the activity for the two recreational skiers.

	Skier A				Skier B			
	Davos Day1	Davos Day2	Zermatt	TOTAL	Davos Day1	Davos Day2	Zermatt	TOTAL
*Distance, km*	13.6	16.9	12.8	**43.4**	13.9	16.9	12.3	**43.2**
*Altitude Drop, m*	−3291	−3795	−2619	**−9705**	−3268	−3767	−2595	**−9630**
*Average Speed, m/s*	8.54	8.56	8.95	**8.64**	6.15	7.51	6.94	**6.70**
*Max Speed, m/s*	16.14	22.45	16.33	**22.45**	13.28	1740	13.07	**17.40**
*Duration, h:mm:ss*	0:54:40	0:57:13	0:35:21	**2:27:14**	1:08:12	0:56:44	0:37:04	**2:42:00**

### Processing

#### GNSS processing.

An accurate absolute global position of the GNSS base station was calculated using reference data from the Swiss GNSS Positioning Service AGNES (Federal Office of Topography Swisstopo. Bern. Switzerland) and geodetic post-processing software Justin (Javad, San Jose, CA, USA). For the ground truth dGNSS position and time, solutions were calculated from the GNSS raw data logged at 50 Hz (Galileo, GPS, GLONASS and Beidou) from the base station and the skiers using the post-processing software Terrapos (Field Group, Oslo, Norway) in double-difference carrier-phase kinematic mode with an elevation cut-off angle of 15°. For 3% of the measurements, when the post-processing software was unable to fix ambiguities (integer ambiguities) in the GNSS solution, the data were excluded from the accuracy assessment. Prior to analysis, the dGNSS position data were filtered using a bi-cubic spline function weighing the position output accuracy estimation (RMS) from post-processing [17]. The smartphone GNSS was processed by the unit’s internal GNSS in standalone mode [14,16] and exported via the respective smartphone apps.

#### Geodetic reference frame synchronization.

Initial data processing was performed using MATLAB R2022b (MathWorks, Natick, MA, USA). Reference position information was stored in UTM32N format with northing and easting as axes. The altitude model of the reference data was based on the GRS80 ellipsoid. Horizontal plane position data for each smartphone app were downloaded and converted into the UTM32N coordinate system. This georeferencing system based on the WGS 84 datum utilizes Universal Transverse Mercator (UTM) projection. Zone 32N encompasses the geographic region between 6°E and 12°E longitude and 0°N and 84°N latitude, with its central meridian at 9°E. Latitude and longitude were converted to UTM32N.

#### Time synchronization.

Time data were converted to GPS time by subtracting 1^9^ seconds to match the 32-bit recording format used by the reference system. An 18-second offset caused by the accumulation of leap seconds since 1980 was also accounted for to align GPS time with the reference system's timeline. To ensure synchronization with the dGNSS ground truth data, timestamps from each app were rounded to the nearest 0.02 seconds, corresponding to the 50 Hz sampling frequency of the reference system. Internal app delays caused by differences in processing times among apps were identified using a least sum of squared differences (SSD) approach. This method involved comparing the time-position curves of the reference system and the app data within a central recording window. The time offset yielding the lowest SSD was determined to be the internal delay for each app. These offsets were corrected by adjusting the app timestamps. The calculated delays for each app are summarized in Table 2.

**Table 2:** Mean time offsets from the true GPS time for each app.

App	Mean Offset, s	n
Strava	1.73	16
iSki	−0.25	12
Slopes	0.33	3
Skitude	0.48	5

*n represents the number of data files per app.*

#### Speed determination.

Speed was calculated by interpolating the position data from each mobile app to a uniform frequency of 1 Hz using linear interpolation. Instantaneous speed was determined by calculating the distance travelled between consecutive points, accounting for northing, easting, and app-reported altitude. To improve accuracy, the speed at each position was averaged in both the forward and backward directions. To reduce noise, we applied a 4th-order low-pass Butterworth filter with a normalized cut-off frequency of 0.4, which is equivalent to a cut-off frequency of 0.2 Hz. The time delay introduced by the filter was corrected using a fixed delay adjustment of $0.419/f_{c}\;$, where $f_{c}$ is the cut-off frequency, following the approach described by Manal and Rose [18]. The same filtering technique was applied to the reference data sampled at 50 Hz. For the reference dataset, the normalized cut-off frequency of 0.4 corresponded to a cut-off frequency of 10 Hz, ensuring consistent filtering across the smartphone and dGNSS data.

#### Data inclusion.

Only data recorded during downhill skiing segments were included in the analysis. These segments were identified using an algorithm that filtered the reference altitude data, selecting datapoints where the elevation decreased at a minimum rate of 0.5 m/s. The downhill skiing data identified by the reference system served as a benchmark for comparison with the smartphone app data. This allowed for the calculation of position and speed errors in subsequent analyses.

#### Horizontal plane position and speed error calculation.

To minimize skewness in the error distribution, outliers were identified and removed. Outliers were defined as data points where the horizontal plane position error exceeded three standard deviations from the mean error across all smartphones and apps in accordance with the outer limits of the empirical rule. Each app used its own altitude reference frame, with specifications that were mostly unknown to the user. This lack of standardization prevented the transformation of altitude data into a common reference frame for comparison with the dGNSS ground truth. However, such a transformation was possible for horizontal plane position. The horizontal plane position error for each smartphone app was calculated as the Euclidean distance between the app’s position estimates and the corresponding dGNSS reference data in the horizontal plane. This approach provided a quantitative measure of the error in the horizontal plane position estimates for each app and device. Notably, the horizontal plane position error contains an uncertainty of several decimeters due to the physical offset between the placement of the smartphones on the skier’s body and the placement of the dGNSS unit mounted on the skier’s back. For speed error, the smartphone speed calculated at each time point was compared to the speed calculated from the reference dGNSS data for the same time point, resulting in an error value expressed in meters per second.

#### Statistical analysis.

Statistical analyses were performed using R (version 2024.04.2 + 764) to evaluate the accuracy of the speed and position estimates across various phone models and apps. The primary objective was to assess the distribution of error and identify significant differences between the phone models and apps. To compare the distributions of error between phones and apps, we performed Kolmogorov-Smirnov tests, which are robust for comparing two samples and do not assume normality of the data. The p-values obtained from these tests are reported to indicate significance at an alpha of 0.01. In addition, Cohen’s d values were calculated for each comparison to provide a measure of the magnitude of differences between the groups and to offer a more practical understanding of the error sizes beyond statistical significance, helping to interpret the real-world implications of the observed differences. Effect sizes were categorized as negligible (d < 0.2), small (d ≥ 0.2), medium (d ≥ 0.5), and large (d ≥ 0.8) [19]. Error distributions for position and speed were visualized using histograms to examine the variability across phones and apps. The analysis was performed for all smartphones together and separately by phone model and app. Smartphones were categorized into four groups, enabling comparisons between higher and lower-end models, as well as between Android and iOS devices: high-end Android, high-end iOS, low-end Android, and low-end iOS.

##### Speed and day dependency.

To assess speed dependency and day dependency, error distributions were compared across different speed bins and days using Kolmogorov-Smirnov tests. Significant differences in errors across smartphone models led to using only the high-end iOS smartphones for the sub-analysis of speed dependency, ensuring a more homogeneous dataset.

## Results

### Horizontal plane position error

**Fig 1:**
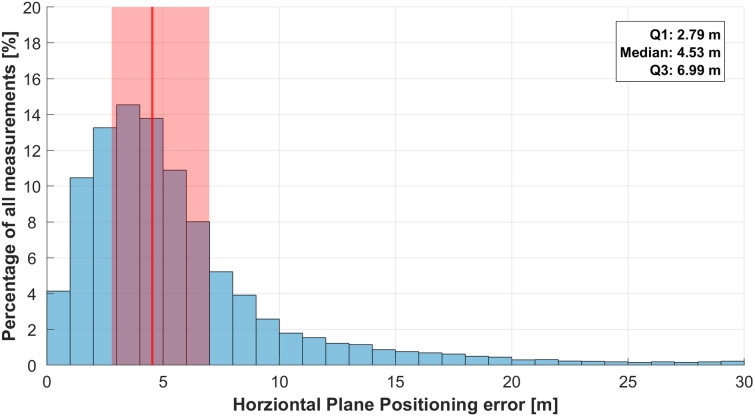
Histogram showing the horizontal plane position error. The median is indicated by a red line and the interquartile range (IQR) as a shaded red area. The histogram bins have a width of 1 m.

The median horizontal plane position error across all data collection days, smartphones, and apps was 4.53 m, with an interquartile range (IQR) of 4.20 m (Fig 1).

#### Horizontal plane position error per smartphone.

**Fig 2:**
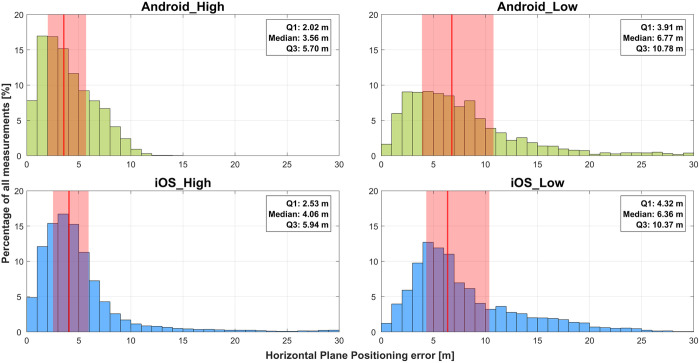
Histograms of the horizontal plane position error of each smartphone model. The medians are indicated as red lines and the interquartile ranges (IQRs) as red shaded areas.

Fig 2 and Table 3 show the horizontal plane position error for each smartphone type. In addition to the analysis of the four smartphone models (*Android_High*, *iOS_High, Android_Low*, and *iOS_Low*), we also tested the low-end models in battery saver mode (*Android_LowBatterySaver*, *iOS_LowBatterySaver*). The results of this testing can be found in S1 File.

**Table 3:** The horizontal plane position errors of the four smartphone models presenting the median, interquartile range (IQR), maximum, minimum, and sample size (n) for horizontal plane positioning errors in meters.

	Median, m	IQR, m	Max, m	Min, m	n
**Android_High**	3.56	3.67	14.04	0.02	3085
**Android_Low**	6.77	6.86	87.21	0.11	5222
**iOS_High**	4.06	3.42	134.72	0.01	25,479
**iOS_Low**	6.36	6.05	129.39	0.24	5880

*IQR, interquartile range; Max, maximum; Min, minimum; n, sample size.*

All pairwise comparisons between the smartphones were significant (p < 0.001; Table 3). Cohen’s d revealed small (0.2 ≤ d < 0.5) effect sizes, with the largest difference between Android_Low and iOS_High (d = 0.354), followed by Android_Low and Android_High (d = 0.342), and iOS_Low and Android_High (d = 0.366). These effect sizes indicate meaningful differences between the high-end and low-end groups, with Android_Low and iOS_Low demonstrating consistently greater horizontal plane position errors (Table 4). No substantial differences were observed between Android and iOS in either the high-end (Android_High vs. iOS_High) or low-end (Android_Low vs. iOS_Low) groups.

**Table 4:** Cohen’s d values for the horizontal plane position error across smartphones displaying Cohen’s d values derived from Kolmogorov–Smirnov tests for all smartphone comparisons. All differences were statistically significant (p < 0.01).

	iOS_High	Android_Low	iOS_Low
**Android_High**	0.062	0.342	0.366
**iOS_High**		0.354	0.349
**Android_Low**			0.063

#### Horizontal plane position error per app.

**Fig 3:**
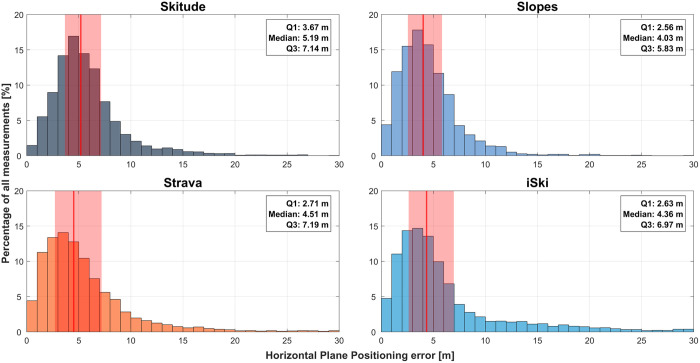
Histograms of the horizontal plane position error with each app (Skitude, Slopes, Strava, iSki). The median is indicated as a red line and the interquartile range (IQR) as a red shaded area.

All pairwise comparisons of position error between the apps were significant (p < 0.01; Table 5 and Fig 3). However, only negligible effect sizes were categorized as negligible (d < 0.2), small (d ≥ 0.2), medium (d ≥ 0.5), and large (d ≥ 0.8) Table 6. The largest effect size was found between Slopes and Skitude (d = 0.197), followed by iSki and Skitude (d = 0.150), and Strava and Skitude (d = 0.136). Overall, the distributions of horizontal plane position error across the app groups were very similar, with only limited differences in effect size.

**Table 5:** The horizontal plane position error with the four smartphone apps.

	Median, m	IQR, m	Max, m	Min, m	n
**iSki**	4.36	4.34	95.60	0.03	13,577
**Skitude**	5.19	3.47	87.21	0.11	5284
**Slopes**	4.03	3.28	49.53	0.12	2526
**Strava**	4.51	4.48	134.72	0.01	18,279

*IQR, interquartile range; Max, maximum; Min, minimum; n, sample size.*

**Table 6:** Cohen’s d values for the horizontal plane position error across apps.

	iSki	Slopes	Skitude
**Strava**	0.036	0.120	0.136
**iSki**		0.113	0.150
**Slopes**			0.197

*Cohen’s d values were derived from Kolmogorov–Smirnov tests for all app comparisons. All differences were significant (p < 0.01).*

#### Speed dependency of position error.

**Fig 4:**
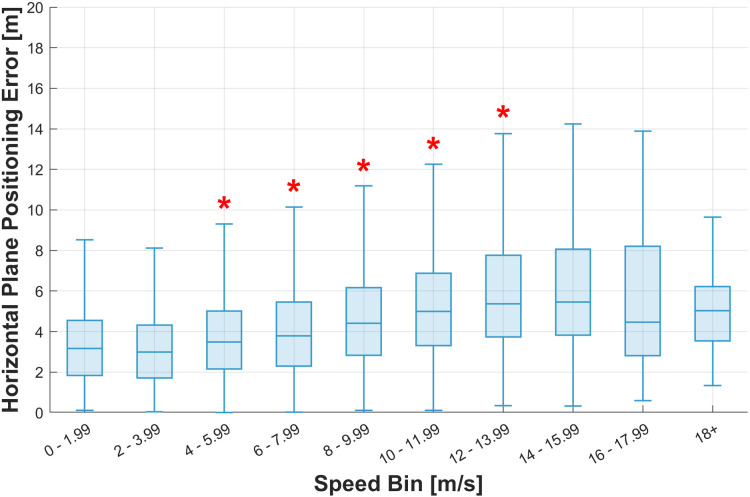
Mean horizontal plane position error across speed bins. The boxplot shows the horizontal plane position error in meters across different speed bins. The boxes depict the interquartile range (IQR), with the median shown as a central line, and whiskers extending to the most extreme data points within 1.5 times the IQR. * Significant differences between the current speed range and the previous one based on the Kolmogorov–Smirnov test (p < 0.01).

Fig 4 depicts the horizontal plane position error for each speed bin, ranging from 0 to 18 + m/s with a bin size of 2 m/s. As shown in Table 7, the comparison of each speed with the previous speed bin was significant, with significant increases in median error observed at several speed transitions.

**Table 7:** Horizontal plane position error by speed bin.

Speed bin	Median, m/s	IQR, m/s	Max, m/s	Min, m/s	n	p-value*
0-1.99 m/s	3.16	2.71	0.12	36.17	739	N/A
2-3.99 m/s	3.00	2.60	0.04	75.37	1170	0.178
**4-5.99 m/s**	**3.48**	**2.87**	**0.01**	**85.30**	**4574**	**<0.000**
**6-7.99 m/s**	**3.78**	**3.14**	**0.03**	**118.22**	**7805**	**<0.000**
**8-9.99 m/s**	**4.41**	**3.34**	**0.11**	**134.72**	**5490**	**<0.000**
**10-11.99 m/s**	**4.99**	**3.58**	**0.12**	**38.74**	**3434**	**<0.000**
**12-13.99 m/s**	**5.36**	**4.03**	**0.35**	**39.12**	**1625**	**<0.000**
14-15.99 m/s	5.47	4.26	0.32	40.85	501	0.804
16-17.99 m/s	4.46	5.40	0.59	17.25	79	0.058
18 + m/s	5.03	2.67	1.34	16.16	62	0.230

*Kolmogorov–Smirnov test comparing to the previous speed bin. Significant differences are bolded. IQR, interquartile range; Max, maximum; Min, minimum; n, sample size; N/A, not available.

The median horizontal plane position error generally increased with speed. A significant increase in the median error of 16.0% was observed between the 2–3.99 m/s bin and the 4–5.99 m/s bin. Subsequent speed bins also showed significant increases: 8.6% between 4–5.99 m/s and 6–7.99 m/s, 18.4% between 6–7.99 m/s and 8–9.99 m/s, 13.1% between 8–9.99 m/s and 10–11.99 m/s, and 7.4% between 10–11.99 m/s and 12–13.99 m/s.

No significant differences were found between the 14–15.99 m/s and 16–17.99 m/s bins, or between the 16–17.99 m/s and 18 + m/s bins, suggesting minimal changes in the horizontal plane position error at a speed >14 m/s. However, at speeds <4 and >14 m/s, the speed bins had fewer data points compared to the other bins that were significantly different.

#### Day dependency and geographic location.

**Fig 5:**
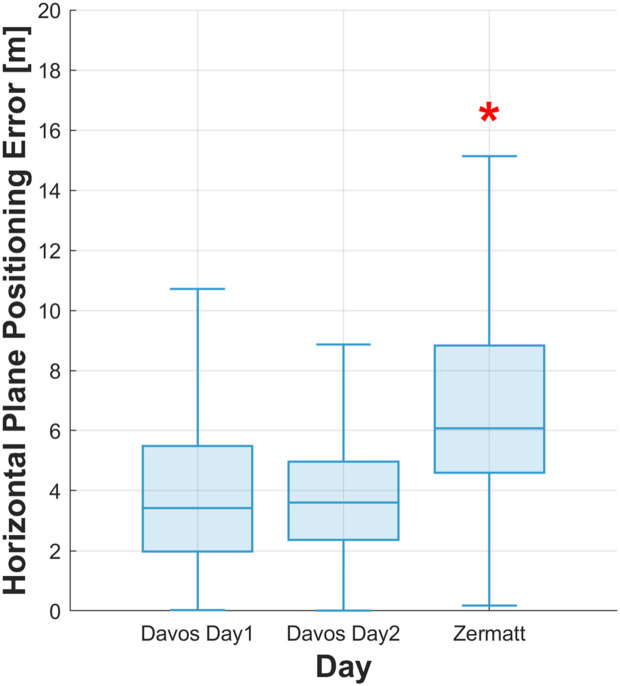
Boxplot of the horizontal plane position error across the three data collection days. The boxes depict the interquartile range (IQR), with the median shown as a central line, and whiskers extending to the most extreme data points within 1.5 times the IQR.

The horizontal plane position error across three data collection days at the two sites is illustrated in Fig 5 and Table 8. Significant location-dependent differences were observed in the horizontal plane position error (Table 9).

**Table 8:** Horizontal plane position error across three data collection days.

	Median, m	IQR, m	Max, m	Min, m	n
**Davos Day1**	3.42	3.51	134.72	0.03	8490
**Davos Day2**	3.60	2.60	26.14	0.01	11387
**Zermatt**	6.07	4.24	34.86	0.17	5602

*IQR, interquartile range; Max, maximum; Min, minimum; n, sample size.*

**Table 9:** Cohen’s d values for the horizontal plane position error across days.

	Davos Day2	Zermatt
**Davos Day1**	0.080	0.426
**Davos Day2**		0.447

*Cohen’s d values were derived from Kolmogorov–Smirnov tests for all day comparisons. All differences were significant (p < 0.01).*

Between Davos Day1 and Davos Day2, we observed a negligible effect size (Cohen’s d = 0.080), with the median position error increasing slightly from 3.42 m on Day 1 to 3.60 m on Day 2. In contrast, medium effect sizes were found when comparing Davos Day1 to Zermatt (d = 0.426) and Davos Day2 to Zermatt (d = 0.447), reflecting substantial increases in the horizontal plane position error. The median error at Zermatt (6.07 m) was significantly higher than on either Davos day, indicating that the surroundings in which the measurement was made significantly influenced the position error.

### Speed error

**Fig 6:**
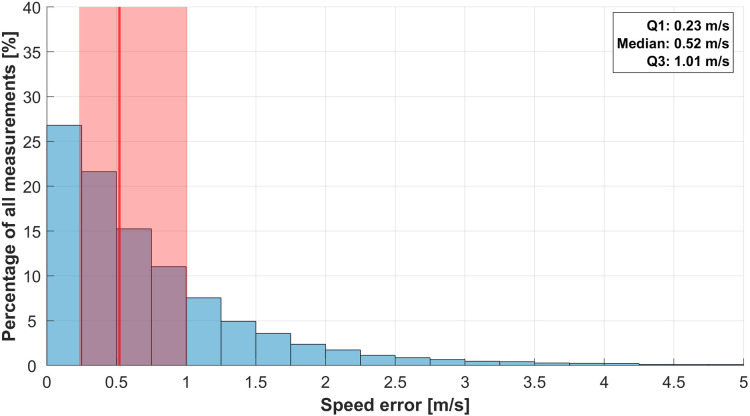
Histogram of the speed error for all smartphones. The median is shown as a red line and the interquartile range (IQR) as a red shaded area. The histogram bins have a width of 0.25 m/s.

The median horizontal plane speed error for all smartphones was 0.52 m/s, with an IQR of 0.78 m/s (Fig 6).

#### Speed error per smartphone.

**Fig 7:**
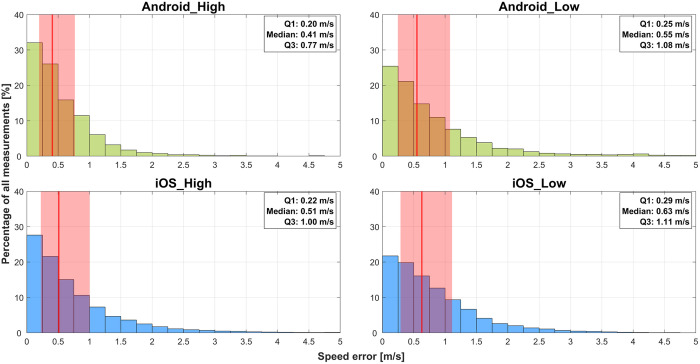
Histograms of the speed error of the different smartphone models. The median is indicated by a red line and the interquartile range (IQR) as a red shaded area.

Analysis of the speed error across the four smartphone conditions revealed minimal differences in accuracy (Fig 7). Table 10 presents the descriptive statistics for speed error, whereas Table 11 shows the results of the Kolmogorov–Smirnov test for pairwise comparisons. All comparisons were significant (p < 0.01), with Cohen’s d values indicating negligible effect sizes (all < 0.2). The largest effect size (0.181) was observed between Android_High and iOS_Low, whereas the smallest effect size (0.055) was between Android_Low and iOS_Low. The effect sizes for comparisons involving high-end and low-end devices were similarly negligible, regardless of operating system.

**Table 10:** The speed error for the four smartphone models.

	Median, m/s	IQR, m/s	Max, m/s	Min, m/s	n
**Android_High**	0.41	0.57	5.46	<0.01	3085
**Android_Low**	0.55	0.83	9.28	<0.01	5222
**iOS_High**	0.51	0.78	21.50	<0.01	25479
**iOS_Low**	0.63	0.82	8.40	<0.01	5880

*IQR, interquartile range; Max, maximum; Min, minimum; n, sample size.*

**Table 11:** Cohen’s d values for the speed error across smartphones.

	iOS_High	Android_Low	iOS_Low
**Android_High**	0.086	0.137	0.181
**iOS_High**		0.058	0.106
**Android_Low**			0.055

*Cohen’s d values were derived from Kolmogorov–Smirnov tests for all smartphone comparisons. All differences were significant (p < 0.01).*

#### Speed error per app.

**Fig 8:**
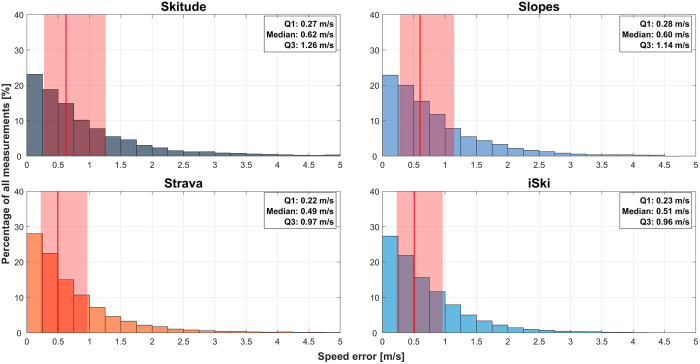
Histograms of the speed error with each app (Skitude, Slopes, Strava, iSki). The median is indicated by a red line and the interquartile range (IQR) by a red shaded area.

Analysis of speed error across the four smartphone apps showed negligible differences in performance. Table 12 and Fig 8 present the descriptive statistics for speed error, and the pairwise comparison results are provided in Table 13. All comparisons were significant (p < 0.01), but Cohen’s d values indicated negligible effect sizes (all < 0.2).

**Table 12:** Speed error with the four smartphone apps.

	Median, m/s	IQR, m/s	Max, m/s	Min, m/s	n
**iSki**	0.51	0.73	20.36	0.00	13577
**Skitude**	0.62	0.98	21.50	0.00	5284
**Slopes**	0.60	0.87	7.80	0.00	2526
**Strava**	0.49	0.75	9.28	0.00	18279

*IQR, interquartile range; Max, maximum; Min, minimum; n, sample size.*

**Table 13:** Cohen’s d values for the speed error across applications.

	iSki	Slopes	Skitude
**Strava**	0.017	0.080*	0.095*
**iSki**		0.069*	0.098*
**Slopes**			0.038

Cohen’s d values were derived from Kolmogorov–Smirnov tests for all application comparisons. * p < 0.01.

#### Speed dependency of speed error.

**Fig 9:**
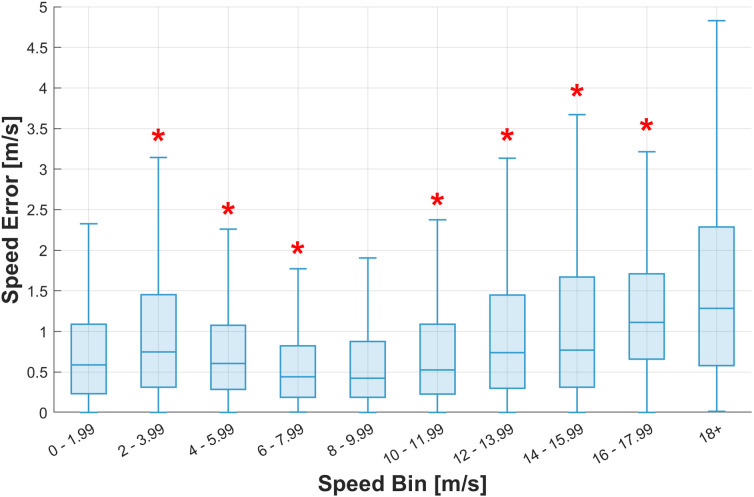
Boxplot of the speed error across the speed range from 0 to 18 + m/s divided into bins of 2 m/s. * Significant differences between a bin and the previous speed bin. The boxes depict the interquartile range (IQR), with the median shown as a central line, and whiskers extending to the most extreme data points within 1.5 times the IQR.

Fig 9 and Table 14 show that the speed error was lowest in the 6–10 m/s speed range. At slower speeds or faster speeds, the error increased.

**Table 14:** Speed error by speed bin.

Speed bin	Median, m/s	IQR, m/s	Max, m/s	Min, m/s	n	p-value*
0-1.99 m/s	0.59	0.86	7.86	<0.00	739	
**2-3.99 m/s**	**0.75**	**1.14**	**7.15**	**<0.00**	**1170**	**<0.001**
**4-5.99 m/s**	**0.61**	**0.79**	**13.94**	**<0.00**	**4574**	**<0.001**
**6-7.99 m/s**	**0.44**	**0.63**	**10.10**	**<0.00**	**7805**	**<0.001**
8-9.99 m/s	0.42	0.69	9.00	<0.00	5490	0.017
**10-11.99 m/s**	**0.53**	**0.86**	**21.50**	**<0.00**	**3434**	**<0.001**
**12-13.99 m/s**	**0.74**	**1.15**	**13.68**	**<0.00**	**1625**	**<0.001**
**14-15.99 m/s**	**0.77**	**1.36**	**14.24**	**<0.00**	**501**	**0.005**
**16-17.99 m/s**	**1.11**	**1.05**	**7.47**	**<0.00**	**79**	**<0.001**
18 + m/s	1.29	1.71	10.19	0.01	62	0.317

IQR, interquartile range; Max, maximum; Min, minimum; n, sample size. *Kolmogorov–Smirnov test. Significant differences from the previous speed bin are in bold.

#### Day and location dependency.

**Fig 10:**
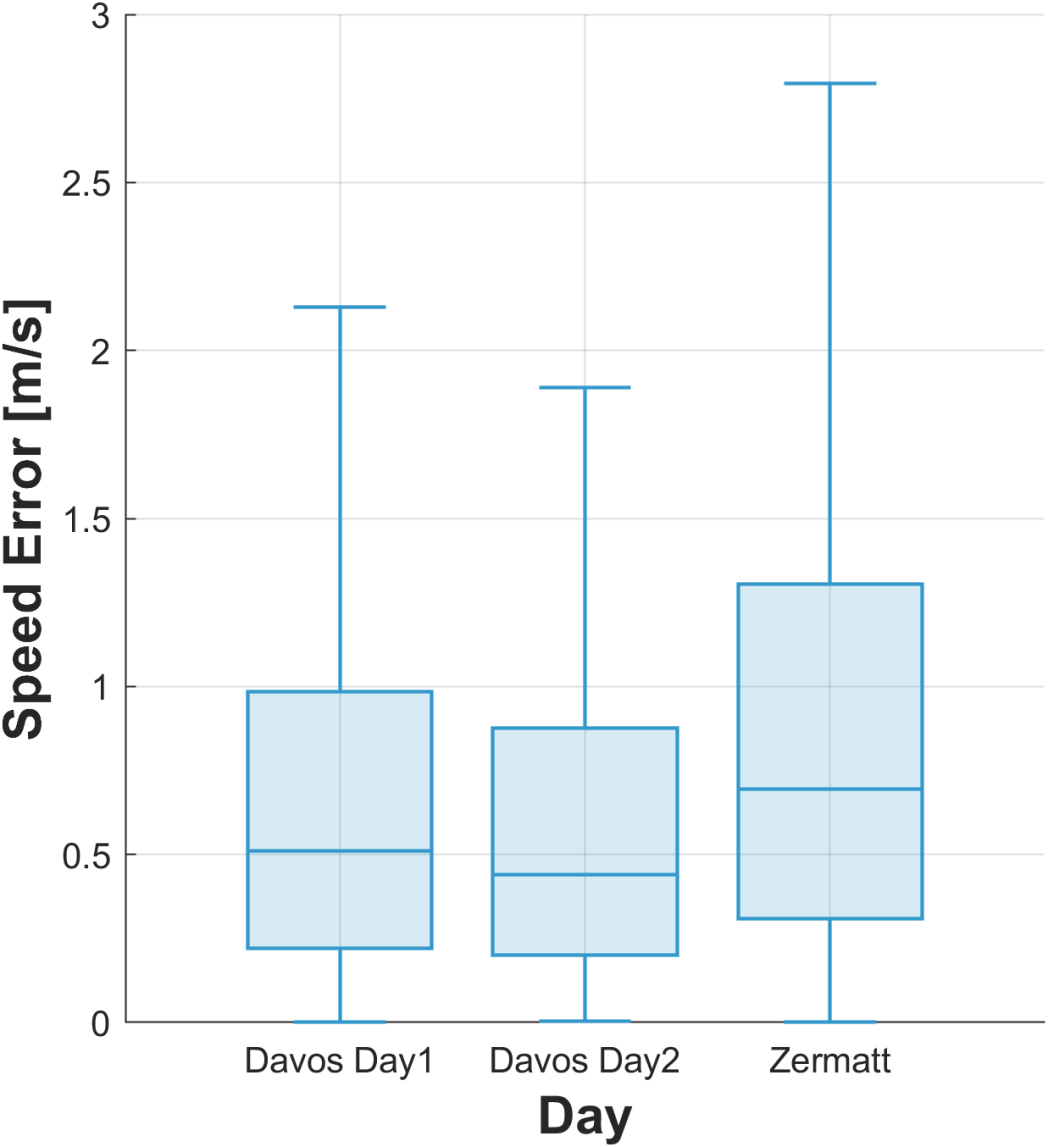
Boxplot of the speed error across the three data collection days. The boxes depict the interquartile range (IQR), with the median shown as a central line, and whiskers extending to the most extreme data points within 1.5 times the IQR.

Analysis of speed error across the different locations (Fig 10) revealed significant differences. Table 15 presents the descriptive statistics for each location, with Davos Day1 having a median error of 0.51 m/s, Davos Day2 0.44 m/s, and Zermatt 0.69 m/s.

**Table 15:** Speed error across the three data collection days at the two locations.

	Median, m/s	IQR, m/s	Max, m/s	Min, m/s	n
Davos Day1	0.51	0.76	13.94	<0.01	8490
Davos Day2	0.44	0.68	14.24	<0.01	11387
Zermatt	0.69	1.00	21.50	<0.01	5602

*IQR, interquartile range; Max, maximum; Min, minimum; n, sample size.*

Table 16 presents the results of the Kolmogorov–Smirnov test for pairwise comparisons between locations. All comparisons were significant (p < 0.01), with Cohen’s d values indicating negligible effect sizes. Differences were present between the locations (Davos and Zermatt) and the largest effect size (0.169) was observed between Davos Day2 and Zermatt. For the same location on different days (Davos Day1 and 2), we found a negligible effect size (0.057). Despite the statistical significance, the practical differences in speed error across locations were minimal.

**Table 16:** Cohen’s d values for the speed error across days.

	Davos Day2	Zermatt
Davos Day1	0.057	0.120
Davos Day2		0.169

*Cohen’s d values were derived from Kolmogorov–Smirnov tests for all day comparisons. All differences were significant (p < 0.01).*

## Discussion

This study demonstrates that the accuracy of motion data collected from smartphones can vary significantly and is influenced by several factors. The quality of the smartphone model is a critical factor in the accuracy, as higher-end devices generally offer more precise measurements. Environmental conditions, such as terrain and the number of available satellites, also have a substantial impact on data accuracy. Furthermore, the speed at which the user moves influences the measurements, with faster speeds potentially introducing greater error.

### Position accuracy

The objective of this study was to assess the accuracy of smartphone GNSS-based position and speed measurements for recreational alpine skiing across four different smartphones and four apps. The results indicate that the median horizontal plane position error for smartphones in alpine skiing apps was 4.2 m, with 75% of all measurements having an error <5.5 m. The majority of the remaining errors, after excluding outliers, were <25 m. High-end smartphones demonstrated superior accuracy for the horizontal plane position error (median ≈ 4 m) compared to low-end models (median ≈ 6.5 m), and the errors were comparable between high-end iOS and high-end Android [19,20]. A limitation of using smartphones to measure position in skiing and snowboarding is the measurement frequency. Consumer-grade team sport GNSS devices operate at 10 Hz [16,21], capturing one position every 1–3 m at typical alpine skiing speeds [3,16,22], enabling a detailed analysis of turns. In contrast, smartphones and their apps usually record positions at 1 Hz, limiting their ability to resolve turns at normal skiing speeds [16,21]. Low-end smartphones had position errors approximately double the error of consumer-grade GNSS for team sports and comparable to 2018/19-era sports watches [21,23]. However, this comparison warrants caution due to differences in testing conditions and generations of technology. Notably, the tests for sports watches were conducted under less dynamic conditions, such as cross-country skiing or on railways to simulate sports motion. These scenarios likely provided a simpler measurement context than alpine skiing, in which the change in the orientation of the upper body of the athlete affects accuracy [16].

Vertical position accuracy was not assessed in this study, as the global reference frames for the vertical position component were not defined in the smartphone app outputs. In addition, whether the altitude is calculated from the GNSS position only or whether barometric data were included in the calculation of the altitude, which has been shown to improve altitude accuracy for certain receivers in mountain running, is not known [24]. However, altitude typically has 2–3-times larger position errors than horizontal plane position [14], and barometers can only improve the relative altitude between points, not the absolute position. Therefore, altitude information with accuracies similar or better than the horizontal plane position may only be obtained through projection of the horizontal plane position onto a digital terrain model with reasonable resolution and terrain model smoothing methodology [25,26].

For smartphones, accuracy on a better scale than meters is not to be expected unless the manner in which position is determined is substantially improved through the use of dual-frequency receivers [11]; the real-time dGNSS app, known as real-time kinematic (RTK) GNSS, where the GNSS data from a base station is sent to the smartphone to enhance its position; or precise point positioning (PPP) technologies. Such technologies are likely to be implemented in smartphones on a large scale in the future [27–30]. Despite the promising results for such technologies, the accuracy range smartphones can reach for dynamic apps needs to be addressed because research has shown that the dynamics of sports hamper GNSS position accuracy compared to static apps for consumer-grade standalone team sports GNSS [20]. For sports watches and team sports consumer-grade standalone GNSS, the literature is inconclusive on the speed dependency of position error [16,21,23]. For the smartphones assessed in this study, speed dependency was present for both position and speed, supporting previous findings that the quality of GNSS receivers has an impact on speed dependency; speed dependency is present with low-end receivers but not industrial grade dGNSS [3,4,31].

Furthermore, the present study found that position accuracy was dependent on the location data collected, with larger errors in Zermatt, where the terrain obstructed the line of sight to satellites to a greater extent than in Davos. These findings align with previous studies of GNSS in alpine skiing, where reduced availability of satellites reduced position accuracy [14].

### Speed accuracy

Speed measurements from smartphones were more accurate than position measurements, with a median error of 0.5 m/s, and 75% of all errors were <1 m/s. This trend is consistent with findings for standalone team sports GNSS devices [16,20,21,32]. The reason for the relatively smaller speed errors compared to position is the type of filtering applied in the GNSS receiver before position is outputted from the GNSS receiver itself, which forces neighboring points to fit well together. As a result, the position–time derivative (speed) is more valid than position, which drifts over time [16,20]. However, the drifting of the position is slow and does not have any substantial effect on speed. The speed errors of the smartphones were substantially larger than the speed errors found for standalone team sports GNSS and sport watches, for which both the central tendency and variation were larger than with the smartphones. This may be caused by both the noise in the position data and the lower measurement frequency of the smartphones compared to the team sports devices, with which time increments are typically 10-times more frequent [16,21]. Despite larger position errors, the slow drift in the position of the smartphones does not significantly affect speed calculations.

Speed accuracy was comparable across high-end and low-end smartphones and unaffected by the choice of smartphone app (iSki, Strava, Slope, or Skitude). These results suggest that speed data from smartphones can be aggregated for population-level analyses, provided that consistent calculation algorithms are used across devices and tracking apps. Therefore, this approach is recommended to reduce the effect of the different speed calculation strategies of the smartphones.

Speed data, though more reliable, also showed slightly increased error at higher speeds, consistent with previous findings that GNSS receiver quality influences speed dependency. These increases were more pronounced in low-end devices, underscoring the importance of considering the dynamics of speed error when aggregating data for analyses.

### Implementation of smartphone apps

This study highlights the significant potential of applying smartphone GNSS data in ski resort planning, slope design, and injury prevention. Speed data are generally more reliable than position data and can provide detailed insights into skier and snowboarder speed distributions based on specific variables, such as slope characteristics, time of day, and weather conditions. For example, aggregated speed data could be used to identify high-speed sections of slopes that may require additional safety measures or modifications to the design.

Although less accurate, position data remain valuable for linking skier or snowboarder locations to slope features, such as steepness, width, and user density. However, these applications require careful consideration of the limitations of the data. As smartphones do not provide estimates of position accuracy, robust outlier detection is essential. Calculating position derivatives can identify periods when changes are unnaturally high [33], such as unrealistic skiing speeds [3,16,22], which may indicate large errors. These outliers should be excluded to prevent misinterpretation, as they can place users far outside the actual slopes. At the beginning of a tracking period or in difficult GNSS conditions, position errors can be very large. Speed accuracy was consistent between smartphones and apps. As speed was calculated as a position-time derivative in this study, it can be used to detect outliers using the same method across smartphones and apps based on the principle that position errors are amplified in the velocity parameter. Thus, large position deviations between neighboring points (in time) can be detected in the speed data as outliers by setting speed thresholds that represent the upper limit of the locomotion mode (e.g., 100 km/h for a recreational skier or 25 km/h for a runner). In addition, geometric projection of smartphone positions onto slope-aligned reference trajectories can mitigate cross-track errors and set reasonable bounds for including data in analyses [21,34]. For high-end smartphones, 75% of horizontal plane position errors were <6 m, whereas low-end devices exhibited errors closer to 10 m. These variations highlight the need for device-specific adjustments in analyses involving position data. [3,16,21,22,33,34]

### Limitations and future research

The purpose of the study was to assess the accuracy of the technology and therefore the number of subjects was limited to two skiers with different skiing characteristics in terms of dynamics and skiing style to cover the influence of skiing style and dynamics on the accuracy of the devices. The technical validation of smartphones conducted in this study is not to be interpreted as an attempt to describe the skiing behavior of the subjects included in this study. Despite promising results, caution is advised when using position data from smartphones for high-precision applications. The accuracy of position data depends on the smartphone model and environmental factors, as errors are amplified by terrain obstructions and speed. Though speed data are more reliable, variations in calculation methods between apps should be considered. Merging data from multiple smartphones and apps requires standardized processing algorithms to ensure comparability. Furthermore, several smartphone apps can be used simultaneously on a single device, as the position data are recorded on the smartphone and accessed from its memory by the apps. Care should be taken during app updates to ensure that changes, such as new smoothing methods, do not influence the data consistency [15,20].

Enhanced technologies, such as RTK GNSS or dual-frequency receivers, could significantly improve GNSS accuracy, particularly for dynamic sports. Further research is needed to evaluate these technologies in real-world skiing conditions. Moreover, the current limitation of the low measurement frequency (1 Hz) of smartphones restricts their ability to accurately capture rapid movements, such as turns and decelerations. Implementing higher-frequency measurements could significantly improve the analysis of dynamic motion in the future. Long-term GNSS data collection presents an opportunity to uncover valuable insights into skier behavior, usage trends, and safety improvements for ski resort planning and management. However, aggregating GNSS data at a population level would introduce critical privacy and security challenges that must be addressed to ensure ethical and secure use of the information. Typically, the terms and conditions of smartphone applications collecting localisation data should explicitly state that such data may be used for scientific research purposes if this is the intention or users should be addressed individually to acquire consent to use their data for such purposes.

## Conclusions

This study demonstrates that smartphone-based GNSS tracking in alpine skiing is affected by several factors, including smartphone quality, environmental conditions, and skier speed. High-end devices, both iOS and Android, consistently outperformed low-end models in terms of accuracy, though significant position errors were still observed. Speed data showed promise for population-level analyses, such as evaluating skiing dynamics and identifying patterns in slope usage. These findings underscore the potential of smartphone GNSS data to contribute to ski resort planning, slope safety enhancements, and injury prevention strategies. However, careful evaluation of data accuracy and the implementation of robust methodologies are needed to maximize its utility.

## Supporting information

S1 File Assessment of battery saver mode in low-end devices. (DOCX)

S1 Data Date, Skier, Phone type, App type, Position error, Speed error. (CSV)
