# Supplementary material for: Accuracy evaluation of smartphone-based GNSS position and speed tracking for ski-slope and safety management
Source: PLoS One. 2025 Aug 13;20(8):e0327896. doi: 10.1371/journal.pone.0327896 (PMC12349078; doi:10.1371/journal.pone.0327896)
Supplement: S1 File — (DOCX) [file pone.0327896.s001.docx]

**S1 File. Assessment of battery saver mode in low-end devices**

The analysis included four smartphone models: high-end Android (Android High), high-end iOS (iOS High), low-end Android (Android Low), and low-end iOS (iOS Low). We tested the low-end models in battery saver mode (Android Low BatterySaver, iOS Low BatterySaver). When comparing positioning errors between the battery saver and standard modes, we observed that the error was smaller in battery saver mode, which was counterintuitive, since it was expected that the battery saving would reduce GNSS accuracy. Given the smaller error and considerably smaller dataset collected in battery saver mode, we decided to merge the data from both modes for consistency in the analysis. This approach assumed that the observed differences in error were likely due to factors other than the battery saver setting itself, as the data for these conditions were not collected simultaneously. Detailed data from the battery saver mode tests are in the figure and table below.


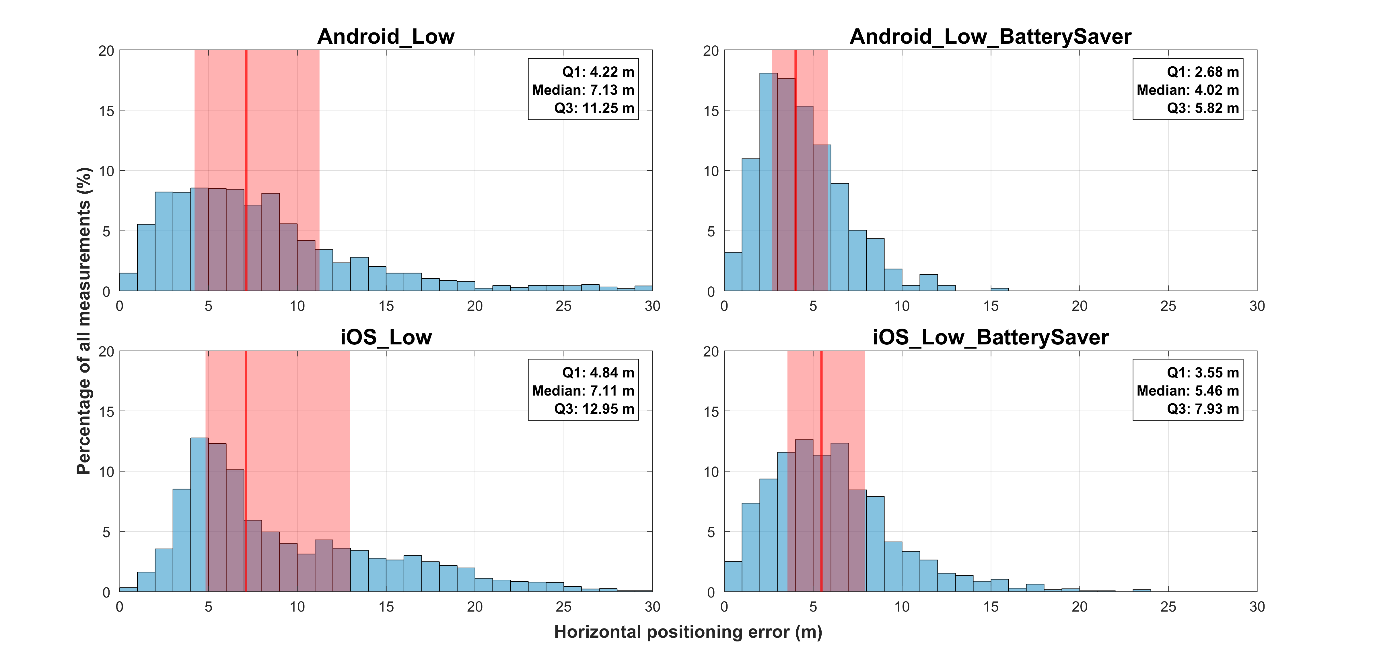


Figure. Histograms of the low-end smartphones in battery saver mode (right) and standard mode (left). Top row, Android. Bottom row, iOS. Each histogram shows the horizontal position error, with the red dashed line representing the median and red shaded area representing the interquartile range (Q1-Q3).

Table. Descriptive statistics for the horizontal plane position errors of the low-end smartphones with and without battery saver mode.

|  | **Median** | **IQR** | **Max** | **Min** | **n** |
| --- | --- | --- | --- | --- | --- |
| Android Low | 7.13 | 7.03 | 87.21 | 0.11 | 4785 |
| Android Low BatterySaver | 4.02 | 3.13 | 15.42 | 0.24 | 437 |
| iOS Low | 7.11 | 8.12 | 129.39 | 0.60 | 3487 |
| iOS Low BatterySaver | 5.46 | 4.38 | 23.14 | 0.24 | 2393 |
